# Supplementary material for: Diagnostic accuracy of the WHO tuberculosis treatment decision algorithms for children with presumptive tuberculosis: An individual participant data meta-analysis
Source: PLoS Med. 2025 Nov 18;22(11):e1004610. doi: 10.1371/journal.pmed.1004610 (PMC12626314; doi:10.1371/journal.pmed.1004610)
Supplement: S1 File — Fig A: WHO treatment decision algorithms (TDAs) adapted from WHO operational handbook on tuberculosis. Module 5: management of tuberculosis in children and adolescents. Geneva: World Health Organization; 2022. Licence: CC BY-NC-SA 3.0 IGO [3]. Table A: Study characteristics of individual studies included in the IPD; TB-Speed decentralisation, TB-Speed HIV, RaPaed-TB and Umoya. Table B: Variable definitions of individual studies included in the IPD; TB-Speed decentralisation, TB-Speed HIV, RaPaed-TB and Umoya. Table C: Outcome definitions used in the study. Fig B: Cascade through TDA A stratified by included study in the IPD. For TB-Speed HIV, this is based on the imputed dataset. WHO TDA images adapted from WHO operational handbook on tuberculosis. Module 5: management of tuberculosis in children and adolescents. Geneva: World Health Organization; 2022. Licence: CC BY-NC-SA 3.0 IGO [3]. Fig C: Cascade through TDA B stratified by included study in the IPD. For TB-Speed HIV, this is based on the imputed dataset. WHO TDA images adapted from WHO operational handbook on tuberculosis. Module 5: management of tuberculosis in children and adolescents. Geneva: World Health Organization; 2022. Licence: CC BY-NC-SA 3.0 IGO [3]. Table D: Diagnostic accuracy of both treatment decision algorithms against a composite reference standard assuming all night sweats in the TB-Speed HIV cohort are absent. Table E: Diagnostic accuracy of both treatment decision algorithms against a composite reference standard assuming all night sweats in the TB-Speed HIV cohort are present. Fig D: Score distribution of children eligible for scoring stratified by site A. TDA A, B. TDA B. (DOCX) [file pmed.1004610.s001.docx]

**Diagnostic accuracy of the WHO tuberculosis treatment decision algorithms for children with presumptive tuberculosis: an individual participant data meta-analysis**

Table of Contents

[Supplemental materials 2](#_Toc211286700)

[Supplemental Figure A: 3](#_Toc211286701)

[Supplemental Table A: Study characteristics of individual studies included in the IPD 4](#_Toc211286702)

[Supplemental Table B: Variable definitions 6](#_Toc211286703)

[Supplemental Table C: Outcome definition used in study 10](#_Toc211286704)

[Supplemental Figure B: Cascade through TDA A stratified by included study in the IPD 11](#_Toc211286705)

[Supplemental Figure C: Cascade through TDA B stratified by included study in the IPD 12](#_Toc211286706)

[Supplemental Table D: Diagnostic accuracy of both treatment decision algorithms against a composite reference standard assuming all night sweats in the TB-Speed HIV cohort are absent 13](#_Toc211286707)

[Supplemental Table E: Diagnostic accuracy of both treatment decision algorithms against a composite reference standard assuming all night sweats in the TB-Speed HIV cohort are present 14](#_Toc211286708)

[Supplemental Figure D: Score distribution of children eligible for scoring stratified by site 15](#_Toc211286709)

[References 16](#_Toc211286710)

## **Supplemental materials**

In the TB-Speed HIV cohort (n=204; 11.9% of the entire IPD), data regarding night sweats was not collected, yet it is part of the score section for both TDAs. To address this issue, we employed a simple imputation strategy based on a decision tree model.

The TB-Speed HIV, RaPaed-TB, and UMOYA children were recruited from tertiary level facilities, whereas TB-Speed Decentralization recruited from district and primary health care centres.

Knowing this, we generated a dataset which includes only the children living with HIV (from studies which are similar in inclusion criteria to TB-Speed HIV) from RaPaed-TB (n=148), UMOYA (n=42), and TB-Speed HIV (n=204); and with the *simputation* package in R (version 0.2.8), we used a classification decision tree approach to predict night sweats status based on clinically relevant variables (age, history of TB contact, severe acute malnutrition, cough ≥ 2 weeks, fever ≥ 2 weeks, weight loss, cavitations on chest X-ray, and Xpert result [Mtb positive or Mtb negative]) using the function *impute_cart()*. The imputed dataset was then used for subsequent analyses, and sensitivity analyses (Supplemental Table 4,5; Supplemental Figures 3,4) were conducted assuming all negative or all positive for nights sweats.

**
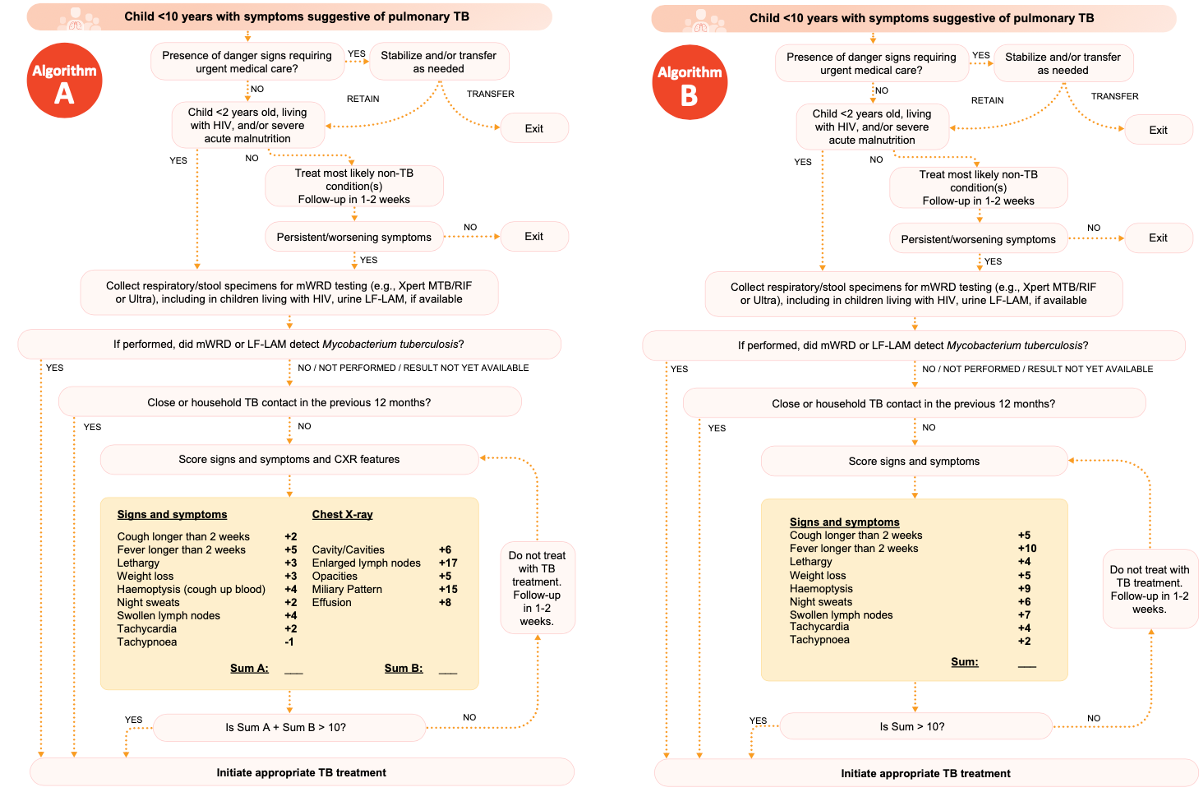
**

## **Supplemental Figure A:**

WHO treatment decision algorithms (TDAs) reproduced from the WHO operational handbook on tuberculosis. Module 5: management of tuberculosis in children and adolescents. Geneva: World Health Organization; 2022. Licence: CC BY 4.0. WHO is not responsible for the content or accuracy of this reproduction.3.

## **Supplemental Table A:** Study characteristics of individual studies included in the IPD

|  | **TB-Speed decentralization** | **TB-Speed HIV** | **RaPaed-TB^2^** | **UMOYA^3^** |
| --- | --- | --- | --- | --- |
| **Aim** | To assess the impact of implementing decentralized diagnostic approaches on childhood TB case detection at district hospital (DH) and primary health centers (PHCs) and to compare decentralization strategies | To externally validate the PAANTHER score in CLHIV with presumptive TB | To evaluate several novel index tests and diagnostic approaches | To i) build a clinical, radiological, and biological sample repository to evaluate new emerging diagnostic tools and biomarkers and ii)) investigate the short and long-term impact of pulmonary TB and other respiratory illnesses on lung health and QoL |
| **Design** | Cross-sectional operational research study with nested prospective cohort | Prospective diagnostic cohort for external validation of TDA | Prospective single-gate diagnostic accuracy study | Prospective observational cohort study |
| **Population** | Children <15 years with presumptive TB | CLHIV <15 years with presumptive TB | Children < 15 years with presumptive TB | Children < 13 years with presumptive TB |
| **Duration** | During study intervention period (March 7, 2020 to September 30, 2021), we  decentralized the comprehensive childhood TB diagnosis  package and documented TB diagnosis. The exact start and end dates varied  across countries. | Participant recruitment was conducted between October 2, 2019 and December 31, 2021 | Participant recruitment was conducted between January 21, 2019, and July 1, 2021 | Participant recruitment was conducted from November 2017 to May 2023 |
| **Setting** | Cambodia, Cameroon, Côte d’Ivoire, Mozambique, Sierra Leone, Uganda | Côte d’Ivoire, Mozambique, Uganda, Zambia | South Africa, Mozambique, Malawi, India, Tanzania | South Africa |
| **Number screened** | 584 | 713 | 5313 | 1330 |
| **Number recruited** | 3,104 / 584 | 277 | 975 | Up to 600 children with presumptive pulmonary TB and 100 healthy controls |
| **Inclusion criteria** | For assessing decentralized diagnostic: Sick children seeking care at OPD of DH or PHC, age <15 years  For comparing decentralization strategies: Age <15 years, Presumptive TB, Presumptive TB identified by the site clinician irrespective of the above criteria, especially presumed extra-pulmonary TB cases, Informed consent signed, Child’s assent obtained in those aged >7 years | Children aged 1 month to 14 years, Documented HIV-infection, presumptive TB, Informed consent signed by parent/guardian | Consent and assent (if applicable), confirmation of TB disease and/or signs and symptoms suggestive of TB | Consent and assent (if applicable), <13 years, > 2.5 kg, presumptive pulmonary TB |
| **Exclusion criteria** | For assessing decentralized diagnostic: None  For comparing decentralization strategies: Children who have received TB treatment in the past 6 months | Ongoing TB treatment or history of intake of anti-TB drugs in the last 3 months (isoniazid alone or rifampin/isoniazid for preventive therapy is not an exclusion criteria) | Critical condition posing an undue risk to the child, body weight < 2 Kg, age ≥ 15 years, currently receiving anti-TB drugs | Extra-thoracic TB only, TB treatment for >2 days in previous 2 weeks, Clear alternative diagnosis at baseline, Severe illness resulting in unstable clinical condition, Contra-indication to sampling procedures, Discharge before baseline sampling completed, Unstable social circumstances, Residence in remote areas |
| **Definition of presumptive TB** | Cough with a duration of >2 weeks, Fever with a duration of >2 weeks, Documented weight loss, History of TB contact with any duration of cough | History of contact with a TB case and any of the symptoms with a shorter duration (< 2 weeks):  Persistent cough for more than 2 weeks, persistent fever for more than 2 weeks, recent failure to thrive (documented clear deviation from a previous growth trajectory in the last 3 months or Z score weight/age < 2), failure of broad-spectrum antibiotics for treatment of pneumonia, suggestive CXR features | - 1. Children with recent MTB confirmation, suggestive CXR findings, history of cough >14 days, any cough combined with weight loss, or signs and symptoms of EPTB | All children with either 1) a short history of respiratory symptoms (<14 days) with either a TB contact, sign of TB infection or abnormal CXR findings or 2) children with a typical presentation for TB of >14 days respiratory symptoms |
| **Chest X-ray review for WHO TDA A scoring** | CXRs were reviewed by an expert review panel until consensus was achieved | CXRs were reviewed by an expert review panel until consensus was achieved | CXRs were reviewed by single expert (HSS) and included features that were scorable for the WHO TDA A scoring. | CXRs were reviewed by single expert (HSS) and included features that were scorable for the WHO TDA A scoring. |

Abbreviations: OPD: outpatient department, PHC; primary health care, DH: district health level

## **Supplemental Table B:** Variable definitions

| **TDA variable** | **WHO TDA definitions (if applicable)** | **RaPaed** | **UMOYA** | **TB-Speed HIV** | **TB-Speed Decentralisation** |
| --- | --- | --- | --- | --- | --- |
| Age/age group |  | Date of birth or age (years) | Date of birth or age (years) | Date of birth or age (years) | Date of birth or age (years) |
| Cough ≥ 2 weeks | Persistent, unremitting cough for 2 weeks or more | Presence of cough at enrolment (binary) and cough duration | Presence of cough at enrolment and classification into acute (incl. number of days) or chronic (>2 weeks) | Presence of cough in the 4 weeks preceding enrolment (binary) and duration | Presence of cough > 2 weeks at enrolment (binary) |
| Weight loss | More than 5% reduction in weight compared with the highest weight recorded in the past 3 months, or failure to thrive (clear deviation from previous growth trajectory, or documented crossing of percentile lines in the preceding 3 months, or WFA Z-score of −2 or less, or weight-for-height Z-score of −2 or less in the absence of information on previous or recent growth trajectory) | Presence of weight loss at enrolment (binary) | Presence of weight loss at enrolment (binary) | Presence of weight loss in the 4 weeks preceding enrolment (binary) and duration | Presence of weight loss at enrolment (binary) |
| Fever ≥ 2 weeks | Persistent fever for 2 weeks or more (the score in the algorithm is based on the duration of fever as per the history rather than the actual temperature on examination) | Presence of fever at enrolment (binary) and duration of fever | Presence of fever at enrolment (binary) and duration of fever | Presence of fever in the 4 weeks preceding enrolment (binary) and duration | Presence of fever > 2 weeks at enrolment (binary) |
| Lethargy | Persistent unexplained lethargy or decrease in playfulness or activity reported by the parent or caregiver | Presence of fatigue at enrolment (binary) | Reduced activity at enrolment (options: normal, reduced playfulness, lethargic) | Presence of fatigue or loss of playfulness in the 4 weeks preceding enrolment (binary) and duration | Presence of lethargy or fatigue, or reduced playfulness at enrolment (binary) |
| Haemoptysis | Expectoration of blood or blood-tinged sputum. This is a very rare symptom in children aged under 10 years and should be distinguished carefully from blood brought up by a child following a nosebleed | Presence of haemoptysis at enrolment (binary) | Presence of haemoptysis at enrolment (binary) | Presence of haemoptysis in the 4 weeks preceding enrolment (binary) and duration | Specification on presence of cough indicating that blood was produced |
| Night sweats | Excessive night-time sweating that soaks the bed or clothes | Presence of night sweats at enrolment (binary) | Presence of night sweats at enrolment (binary) | **Missing/not collected** | Presence of night sweats at enrolment (binary) only in children ≥ 5 years |
| Swollen lymph nodes | Non-painful, enlarged cervical, submandibular or axillary lymph nodes | Presence of lymph node at enrolment (binary) which are not characterised as painful, are bigger than 1cm, and are cervical, axillar, or submandibular | Presence of lymphadenopathy at enrolment (binary) which are bigger than 1.5cm, and are cervical, axillar, or submandibular | Presence of cervical or supra-clavicular Adenopathy (no, single, multiple) | Presence of adenopathy at enrolment (binary) with specified location (supra-clavicular or cervical), number, and size of biggest node (1-3 cm or >3 cm) |
| Tachycardia | Children aged under 2 months: heart rate over 160 beats/minute  Children aged 2–12 months: heart rate over 150 beats/minute  Children aged 12 months to 5 years: heart rate over 140 beats/minute  Children aged over 5 years: heart rate over 120 beats/minute | Children aged under 2 months: heart rate over 160 beats/minute  Children aged 2–12 months: heart rate over 150 beats/minute  Children aged 12 months to 5 years: heart rate over 140 beats/minute  Children aged over 5 years: heart rate over 120 beats/minute | Children aged under 2 months: heart rate over 160 beats/minute  Children aged 2–12 months: heart rate over 150 beats/minute  Children aged 12 months to 5 years: heart rate over 140 beats/minute  Children aged over 5 years: heart rate over 120 beats/minute | Children aged under 1 year: heart rate over 160 beats/minute  Children aged 1–2 years: heart rate over 150 beats/minute  Children aged 2-5 years: heart rate over 140 beats/minute  Children aged over 5 years: heart rate over 120 beats/minute | Children aged under 2 months: heart rate over 160 beats/minute  Children aged 2–12 months: heart rate over 150 beats/minute  Children aged 12 months to 5 years: heart rate over 140 beats/minute  Children aged over 5 years: heart rate over 120 beats/minute |
| Tachypnoea | Children aged under 2 months: respiratory rate over 60/minute  Children aged 2–12 months: respiratory rate over 50/minute  Children aged 12 months to 5 years: respiratory rate over 40/minute  Children aged over 5 years: respiratory rate over 30/minute | Children aged under 2 months: respiratory rate over 60/minute  Children aged 2–12 months: respiratory rate over 50/minute  Children aged 12 months to 5 years: respiratory rate over 40/minute  Children aged over 5 years: respiratory rate over 30/minute | Children aged under 2 months: respiratory rate over 60/minute  Children aged 2–12 months: respiratory rate over 50/minute  Children aged 12 months to 5 years: respiratory rate over 40/minute  Children aged over 5 years: respiratory rate over 30/minute | Not collected but respiratory rate and age available | Children aged under 2 months: respiratory rate over 60/minute  Children aged 2–12 months: respiratory rate over 50/minute  Children aged 12 months to 5 years: respiratory rate over 40/minute  Children aged over 5 years: respiratory rate over 30/minute |
| HIV status |  | Overall HIV status generated based on reported status and test results. Overall HIV test results based on the combination of the first test with the second and third confirmatory test | Overall HIV status generated based on reported status and test results | Entire cohort is living with HIV based on inclusion criteria for the study | HIV status following testing and in children < 18 months, an additional PCR was done |
| SAM |  | Based on weight for length Z-score and BMI-for-age Z-score < -3 (weight for length used in those under 5) | If weight for length <-3 Z-score or bilateral pitting oedema for those under 5 months of age  If weight for length <-3 Z-score or MUAC<11.5cm (with or without oedema for those over 5 months) | BMI-for-age Z-score < -3 (weight for height in those under 5); MUAC; presence of oedema | Weight for height Z-score < -3 if child aged < 5 years or binary indication of severe acute malnutrition |
| Collected respiratory specimen |  | Sample type indicated as spontaneous or induced sputum, nasopharyngeal aspirate, gastric aspirate, or other respiratory sample | Indication if two respiratory samples were collected and if not, how many were collected | Sample type indicated as expectorated sputum, gastric aspirate or nasopharyngeal aspirate | Indication of expectorated sputum collection and of nasopharyngeal aspirate collection (binary) |
| Collected stool specimen |  | Sample type indicated as stool | Indication of stool collection (binary) | Sample type indicated as stool | Indication of stool collection (binary) |
| Xpert conducted |  | Type of Xpert test (MTB-Rif or MTB Rif Ultra) | Type of Xpert test (MTB-Rif or MTB Rif Ultra) | Type of Xpert test (MTB-Rif or MTB Rif Ultra) | Type of Xpert test (MTB-Rif or MTB Rif Ultra) |
| Urine LAM conducted |  | Child living with HIV indicated LF-LAM (both Alere or FujiLAM) conducted | Not conducted, but urine was collected | Not conducted, but urine was collected | **Missing/not collected** |
| Mtb final result |  | Final Xpert result (positive or negative). This is the highest result as multiple samples were collected per child | Mtb complex detected or trace result | Mtb complex detected or trace result | Mtb complex detected or trace result |
| TB contact < 12 months |  | TB contact at 24 months | Indication of TB contact in the past 12 months | Indication of TB contact in the past 12 months | Indication of TB contact in the past year |
| Cavities |  | Presence of cavities at enrolment evaluated by external reviewers (n=3) | Presence of cavities at enrolment evaluated by external reviewers (n=3) | Presence of cavities at enrolment evaluated by local radiographer (binary) | Presence of cavities at enrolment evaluated by study clinician, radiographer, or clinical officer (indicated) (n=3) |
| Enlarged lymph nodes |  | Presence of enlarged lymph nodes at enrolment evaluated by external reviewers (n=3) | Presence of enlarged lymph nodes at enrolment evaluated by external reviewers (n=3) | Presence of cavities at enrolment evaluated by local radiographer (binary) | Presence of enlarged lymph nodes at enrolment evaluated by study clinician, radiographer, or clinical officer (indicated) (n=3) |
| Opacities |  | Presence of opacities at enrolment evaluated by external reviewers (n=3) | Presence of opacities at enrolment evaluated by external reviewers (n=3) | Presence of interstitial opacities at enrolment evaluated by local radiographer (binary) | Presence of opacities at enrolment evaluated by study clinician, radiographer, or clinical officer (indicated) (n=3) |
| Miliary pattern |  | Presence of miliary patterns at enrolment evaluated by external reviewers (n=3) | Presence of miliary patterns at enrolment evaluated by external reviewers (n=3) | Presence of miliary at enrolment evaluated by local radiographer (binary) | Presence of miliary patterns at enrolment evaluated by study clinician, radiographer, or clinical officer (indicated) (n=3) |
| Effusion |  | Presence of effusion at enrolment evaluated by external reviewers (n=3) | Presence of effusion at enrolment evaluated by external reviewers (n=3) | Presence of pleural, pericardial or peritoneal effusion at enrolment evaluated by local ultrasonographer and of pleural effusion evaluated by local radiographer (binary) | Presence of effusion at enrolment evaluated by study clinician, radiographer, or clinical officer (indicated) (n=3) |
| Diagnostic classification |  | Based on NIH criteria (2015) | Based on NIH criteria (2015) | Based on NIH criteria (2015) | Based on NIH criteria (2015) |

Most variables were able to be used or minorly manipulated to fit the WHO definitions to be used in the TDA. There were two cases where changes had to be made; 1) as TB contact was recorded in variations including no time specification, within 12 months, or within 24 months, for this study we took any TB contact as positive. 2) Night sweats was entirely missing/not collected in the TB-Speed HIV cohort and we thus conducted simple imputation based on a logistic regression to address the missingness.

Abbreviations: SAM: severe acute malnutrition, NIH: National Institutes of Health, Mtb: mycobacterium tuberculosis, PCR: polymerase chain reaction, MUAC: mid upper arm circumference, WFA: weight-for-age

## **Supplemental Table C:** Outcome definition used in study

|  | **Categories** | **Definitions** |
| --- | --- | --- |
| TB classification | Unclassifiable | Children that were started on TB treatment and stopped within the first 2 months  Children that were GeneXpert Ultra trace positive but considered infected only (receiving IPT) |
|  | Unlikely | Bacteriological confirmation NOT obtained AND criteria for “unconfirmed tuberculosis” NOT met OR children that met “unconfirmed tuberculosis” but did not receive anti-tuberculosis treatment and were well at follow-up |
|  | Unconfirmed | Bacteriological confirmation NOT obtained AND at least 2 of the following:   - Symptoms/signs suggestive of tuberculosis^1^ (as defined) - Chest radiograph consistent with tuberculosis - Close tuberculosis exposure or immunologic evidence of *Mtb* infection - Positive response to tuberculosis treatment (requires documented positive clinical response on tuberculosis treatment—no time duration specified) |
|  | Confirmed | Bacteriological confirmation obtained - requires *Mtb* to be confirmed (culture, Xpert MTB/RIF, or Xpert MTB/Rif Ultra assay) from at least 1 respiratory specimen |

^1^Symptoms/signs suggestive of TB include cough for more than 2 weeks, fever for more than 2 weeks, and poor weight gain or weight loss in the past 3 months. In young children (< 5 years), reduced playfulness or lethargy is also included

^2^Chest X-rays were assessed by study-specific clinicians or external experts and defined as “attributable to TB”, “unlikely TB”, or “normal”. According to the operational handbook, common abnormalities in children include enlarged hilar and paratracheal lymph nodes, alveolar consolidation without visible cavities, miliary lesions, and pleural effusions.^1^

Abbreviations: IGRA, interferon-γ release assay; TST, tuberculin skin test; *Mtb, mycobacterium tuberculosis*.

**
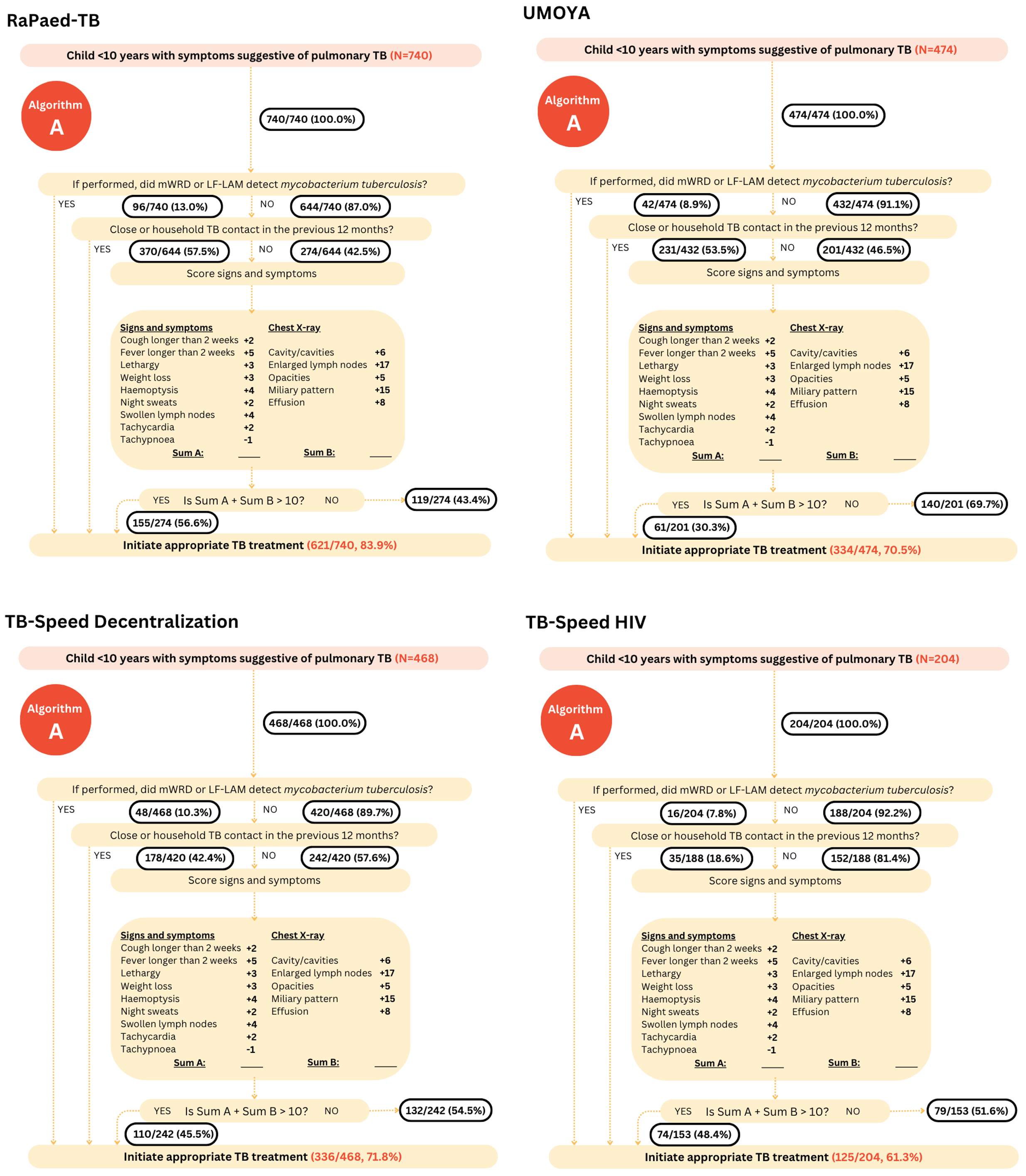
**

## **Supplemental Figure B:** Cascade through TDA A stratified by included study in the IPD

For TB-Speed HIV, this is based on the imputed dataset. WHO TDA images adapted from WHO operational handbook on tuberculosis. Module 5: management of tuberculosis in children and adolescents. Geneva: World Health Organization; 2022. Licence: CC BY 4.0. WHO is not responsible for the content or accuracy of this adaptation.^3^

**
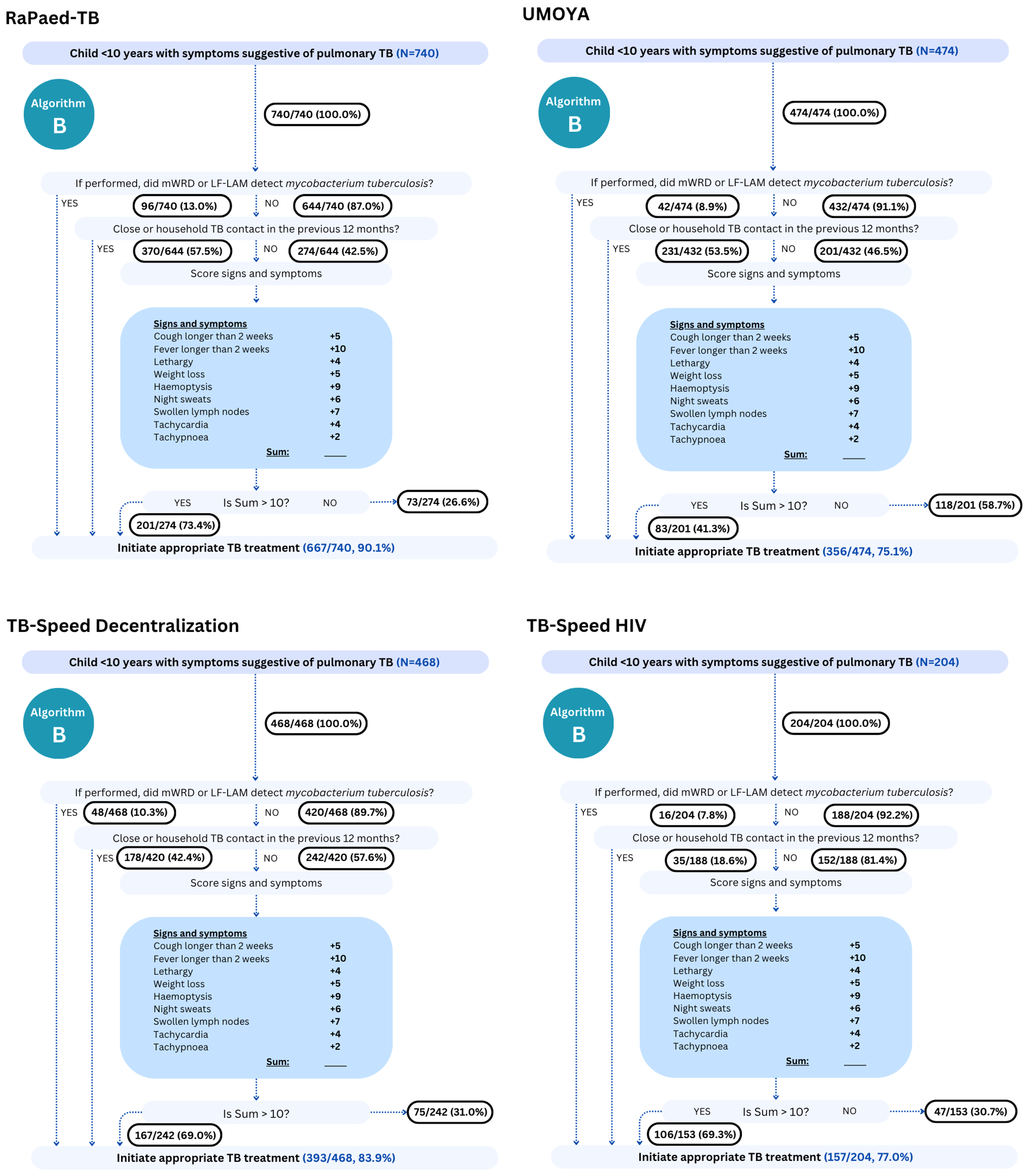
**

## **Supplemental Figure C:** Cascade through TDA B stratified by included study in the IPD

For TB-Speed HIV, this is based on the imputed dataset. WHO TDA images adapted from WHO operational handbook on tuberculosis. Module 5: management of tuberculosis in children and adolescents. Geneva: World Health Organization; 2022. Licence: CC BY 4.0. WHO is not responsible for the content or accuracy of this adaptation.^3^

## **Supplemental Table D**: Diagnostic accuracy of both treatment decision algorithms against a composite reference standard assuming all night sweats in the TB-Speed HIV cohort are absent

| **Subgroup** | **TP** | **FP** | | **FN** | | **TN** | **Sensitivity** | | **Specificity** | |
| --- | --- | --- | --- | --- | --- | --- | --- | --- | --- | --- |
| **TDA A** | | | | | | | | | | |
| **Pooled^#^** | 812 | 541 | | 141 | | 392 | **86.4%** | **(76.4% - 92.6%)** | **43.7%** | **(17.7% - 61.2%)** |
| **Study** |  |  | |  | |  |  |  |  |  |
| RaPaed | 323 | 280 | | 41 | | 96 | 88.7% | (85.1% - 91.6%) | 25.5% | (21.4% - 30.2%) |
| UMOYA | 182 | 143 | | 35 | | 114 | 83.9% | (78.4% - 88.1%) | 44.4% | (38.4% - 50.5%) |
| TB-Speed Dec. | 225 | 97 | | 25 | | 121 | 90.0% | (85.6% - 93.1%) | 55.5% | (48.9% - 62.0%) |
| TB-Speed HIV | 82 | 21 | | 40 | | 61 | 67.2% | (58.4% - 74.9%) | 74.4% | (63.9% - 82.5%) |
| **Risk group** | | |  | |  |  |  |  |  |  |
| High | 393 | 184 | | 84 | | 186 | 82.4% | (78.7% - 85.5%) | 50.3% | (45.2% - 55.3%) |
| Low | 419 | 357 | | 57 | | 206 | 88.0% | (84.8% - 90.6%) | 36.6% | (32.7% - 40.7%) |
| **HIV status** |  | |  | |  |  |  |  |  |  |
| HIV negative | 636 | 470 | | 81 | | 289 | 88.7% | (86.2% - 90.8%) | 38.1% | (34.7% - 41.6%) |
| HIV positive | 170 | 58 | | 60 | | 94 | 73.9% | (67.9% - 79.2%) | 61.8% | (53.9% - 69.2%) |
| Unknown | 6 | 13 | | 0 | | 9 | 100.0% | (56.8% - 93.4%) | 40.9% | (23.4% - 61.4%) |
| **Malnutrition** | | |  | |  |  |  |  |  |  |
| No SAM | 668 | 484 | | 112 | | 338 | 85.6% | (83.0% - 87.9%) | 41.1% | (37.8% - 44.5%) |
| SAM | 144 | 57 | | 29 | | 54 | 83.2% | (76.9% - 88.0%) | 48.6% | (39.6% - 57.8%) |
| **Age** |  | |  | |  |  |  |  |  |  |
| < 2 years | 337 | 163 | | 66 | | 175 | 83.6% | (79.7% - 86.9%) | 51.8% | (46.5% - 57.1%) |
| 2 - < 5 years | 247 | 182 | | 47 | | 120 | 84.0% | (79.4% - 87.7%) | 39.7% | (34.4% - 45.4%) |
| 5 - <10 years | 228 | 196 | | 28 | | 97 | 89.1% | (84.6% - 92.3%) | 33.1% | (28.0% - 38.7%) |
| **TDA B** | | | | | | | | | | |
| **Pooled^#^** | 867 | 665 | | 86 | | 268 | **91.5%** | **(84.4% - 95.6%)** | **27.7%** | **(18.4% - 39.4%)** |
| **Study** |  | |  | |  |  |  |  |  |  |
| RaPaed | 343 | 307 | | 21 | | 69 | 94.2% | (91.3% - 96.2%) | 18.4% | (14.8% - 22.6%) |
| UMOYA | 184 | 162 | | 33 | | 95 | 84.8% | (79.4% - 88.9%) | 37.0% | (31.3% - 43.0%) |
| TB-Speed Dec. | 237 | 149 | | 13 | | 69 | 94.8% | (91.3% - 96.9%) | 31.7% | (25.9% - 38.1%) |
| TB-Speed HIV | 103 | 47 | | 19 | | 35 | 84.4% | (76.9% - 89.7%) | 42.7% | (32.6% - 53.5%) |
| **Risk group** | | |  | |  |  |  |  |  |  |
| High | 420 | 240 | | 57 | | 130 | 88.1% | (84.8% - 90.7%) | 35.1% | (30.5% - 40.1%) |
| Low | 447 | 425 | | 29 | | 138 | 93.9% | (91.4% - 95.7%) | 24.5% | (21.1% - 28.2%) |
| **HIV status** |  | |  | |  |  |  |  |  |  |
| HIV negative | 660 | 550 | | 57 | | 209 | 92.1% | (89.8% - 93.8%) | 27.5% | (24.5% - 30.8%) |
| HIV positive | 201 | 98 | | 29 | | 54 | 87.4% | (82.5% - 91.0%) | 35.5% | (28.4% - 43.4%) |
| Unknown | 6 | 17 | | 0 | | 5 | 100.0% | (56.8% - 93.4%) | 22.7% | (10.6% - 43.9%) |
| **Malnutrition** | | |  | |  |  |  |  |  |  |
| No SAM | 711 | 579 | | 69 | | 243 | 91.2% | (89.0% - 92.9%) | 29.6% | (26.6% - 32.8%) |
| SAM | 156 | 86 | | 17 | | 25 | 90.2% | (84.8% - 93.7%) | 22.5% | (15.8% - 31.2%) |
| **Age** |  | |  | |  |  |  |  |  |  |
| < 2 years | 355 | 208 | | 48 | | 130 | 88.1% | (84.6% - 90.9%) | 38.5% | (33.4% - 43.8%) |
| 2 - < 5 years | 266 | 218 | | 28 | | 84 | 90.5% | (86.6% - 93.3%) | 27.8% | (23.1% - 33.1%) |
| 5 - <10 years | 246 | 239 | | 10 | | 54 | 96.1% | (92.9% - 97.8%) | 18.4% | (14.4% - 23.3%) |

^#^ To account for heterogeneity between studies, a random-effects meta-analysis was conducted for the pooled estimate (R package *mada* [*reitsma* function])

Abbreviations: SAM: severe acute malnutrition, TB-Speed Dec.: TB-Speed Decentralization

## **Supplemental Table E**: Diagnostic accuracy of both treatment decision algorithms against a composite reference standard assuming all night sweats in the TB-Speed HIV cohort are present

| **Subgroup** | **TP** | **FP** | | **FN** | | **TN** | **Sensitivity** | | **Specificity** | |
| --- | --- | --- | --- | --- | --- | --- | --- | --- | --- | --- |
| **TDA A** | | | | | | | | | | |
| **Pooled^#^** | **858** | **601** | | **78** | | **317** | **92.8%** | **(86.1% - 96.4%)** | **34.7%** | **(23.3% - 48.1%)** |
| **Study** |  |  | |  | |  |  |  |  |  |
| RaPaed | 318 | 281 | | 29 | | 80 | 91.6% | (88.2% - 94.1%) | 22.2% | (18.2% - 26.7%) |
| UMOYA | 183 | 151 | | 34 | | 106 | 84.3% | (78.9% - 88.5%) | 41.2% | (35.4% - 47.4%) |
| TB-Speed Dec. | 239 | 110 | | 11 | | 108 | 95.6% | (92.2% - 97.5%) | 49.5% | (43.0% - 56.1%) |
| TB-Speed HIV | 118 | 59 | | 4 | | 23 | 96.7% | (91.7% - 98.6%) | 28.0% | (19.5% - 38.6%) |
| **Risk group** | | |  | |  |  |  |  |  |  |
| High | 428 | 222 | | 44 | | 144 | 90.7% | (87.7% - 93.0%) | 39.3% | (34.5% - 44.4%) |
| Low | 430 | 379 | | 34 | | 173 | 92.7% | (89.9% - 94.7%) | 31.3% | (27.6% - 35.3%) |
| **HIV status** |  | |  | |  |  |  |  |  |  |
| HIV negative | 644 | 488 | | 58 | | 261 | 91.7% | (89.5% - 93.6%) | 34.8% | (31.5% - 38.8%) |
| HIV positive | 208 | 99 | | 20 | | 50 | 91.2% | (86.8% - 94.2%) | 33.6% | (26.5% - 41.5%) |
| Unknown | 6 | 14 | | 0 | | 6 | 100.0% | (56.8% - 100.0%) | 30.0% | (14.9% - 52.2%) |
| **Malnutrition** | | |  | |  |  |  |  |  |  |
| No SAM | 687 | 519 | | 69 | | 284 | 90.9% | (88.6% - 92.7%) | 35.4% | (32.1% - 38.7%) |
| SAM | 171 | 82 | | 9 | | 33 | 95.0% | (90.7% - 97.3%) | 28.7% | (21.3% - 37.6%) |
| **Age** |  | |  | |  |  |  |  |  |  |
| < 2 years | 356 | 191 | | 40 | | 139 | 89.9% | (86.5% - 92.5%) | 42.1% | (36.9% - 47.5%) |
| 2 - < 5 years | 266 | 196 | | 25 | | 104 | 91.4% | (87.6% - 94.1%) | 34.4% | (29.3% - 40.0%) |
| 5 - <10 years | 236 | 214 | | 13 | | 75 | 94.8% | (91.2% - 96.9%) | 26.0% | (21.3% - 31.3%) |
| **TDA B** | | | | | | | | | | |
| **Pooled^#^** | **880** | **712** | | **56** | | **206** | **95.5%** | **(87.2% - 98.5%)** | **28.7%** | **(8.7% - 35.6%)** |
| **Study** |  | |  | |  |  |  |  |  |  |
| RaPaed | 333 | 311 | | 14 | | 50 | 96.0% | (93.3% - 97.6%) | 13.9% | (10.7% - 17.8%) |
| UMOYA | 187 | 169 | | 30 | | 88 | 86.2% | (80.9% - 90.1%) | 34.2% | (28.7% - 40.2%) |
| TB-Speed Dec. | 238 | 155 | | 12 | | 63 | 95.2% | (91.8% - 97.2%) | 28.9% | (23.3% - 35.3%) |
| TB-Speed HIV | 122 | 77 | | 0 | | 5 | 100.0% | (96.6% - 100.0%) | 6.1% | (2.9% - 13.7%) |
| **Risk group** | | |  | |  |  |  |  |  |  |
| High | 434 | 269 | | 38 | | 97 | 91.9% | (89.1% - 94.1%) | 26.5% | (22.3% - 31.3%) |
| Low | 446 | 443 | | 18 | | 109 | 96.1% | (93.9% - 97.5%) | 19.7% | (16.7% - 23.3%) |
| **HIV status** |  | |  | |  |  |  |  |  |  |
| HIV negative | 655 | 568 | | 47 | | 181 | 93.3% | (91.2% - 94.9%) | 24.2% | (21.2% - 27.4%) |
| HIV positive | 219 | 129 | | 9 | | 20 | 96.1% | (92.6% - 97.9%) | 13.4% | (8.9% - 19.9%) |
| Unknown | 6 | 15 | | 0 | | 5 | 100.0% | (56.8% - 100.0%) | 25.0% | (11.7% - 47.3%) |
| **Malnutrition** | | |  | |  |  |  |  |  |  |
| No SAM | 707 | 607 | | 49 | | 196 | 93.5% | (91.5% - 95.1%) | 24.4% | (21.6% - 27.5%) |
| SAM | 173 | 105 | | 7 | | 10 | 96.1% | (92.1% - 98.0%) | 8.7% | (4.9% - 15.4%) |
| **Age** |  | |  | |  |  |  |  |  |  |
| < 2 years | 360 | 230 | | 36 | | 100 | 90.9% | (87.7% - 93.3%) | 30.3% | (25.6% - 35.5%) |
| 2 - < 5 years | 277 | 230 | | 14 | | 69 | 95.2% | (92.1% - 97.1%) | 23.1% | (18.7% - 28.2%) |
| 5 - <10 years | 243 | 252 | | 6 | | 37 | 97.6% | (94.8% - 98.8%) | 12.8% | (9.5% - 17.2%) |

^#^ To account for heterogeneity between studies, a random-effects meta-analysis was conducted for the pooled estimate (R package *mada* [*reitsma* function])

Abbreviations: SAM: severe acute malnutrition, TB-Speed Dec.: TB-Speed Decentralization


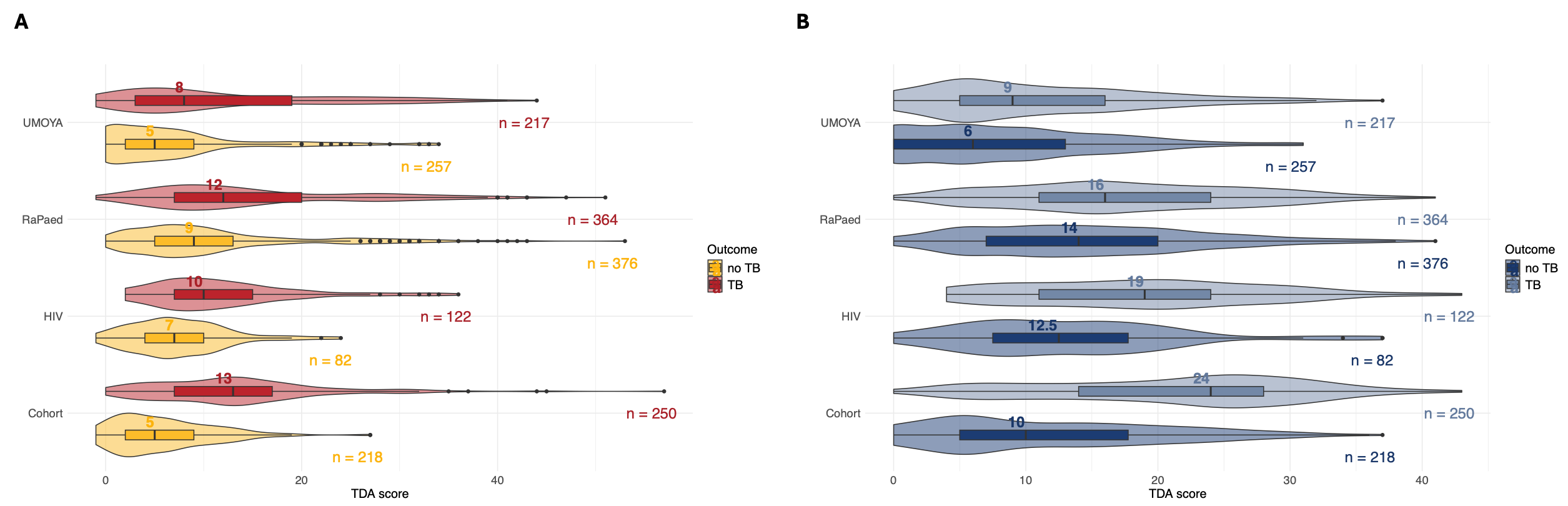


## **Supplemental Figure D:** Score distribution of children eligible for scoring stratified by site

**A.** TDA A, **B.** TDA B.

# References

1. World Health Organization. WHO Operational handbook on tuberculosis: module 5: management of tuberculosis in children and adolescents: World Health Organization; 2022.

2. Olbrich L, Nliwasa M, Sabi I, et al. Rapid and Accurate Diagnosis of Pediatric Tuberculosis Disease: A Diagnostic Accuracy Study for Pediatric Tuberculosis. *Pediatr Infect Dis J* 2023; **42**(5): 353-60.

3. Dewandel I, van Niekerk M, Ghimenton-Walters E, et al. UMOYA: a prospective longitudinal cohort study to evaluate novel diagnostic tools and to assess long-term impact on lung health in South African children with presumptive pulmonary TB—a study protocol. *BMC Pulmonary Medicine* 2023; **23**(1): 97.
