# Supplementary material for: Diagnostic accuracy of the WHO tuberculosis treatment decision algorithms for children with presumptive tuberculosis: An individual participant data meta-analysis
Source: PLoS Med. 2025 Nov 18;22(11):e1004610. doi: 10.1371/journal.pmed.1004610 (PMC12626314; doi:10.1371/journal.pmed.1004610)
Supplement: Attachment — Submitted filename: ResponseToReviewers_20250722.docx [file pmed.1004610.s004.docx]

Cape Town 22 July 2025

Dear Editors and Reviewers,

Thank you for your consideration of our manuscript titled “Diagnostic accuracy of the WHO tuberculosis treatment decision algorithms for children with presumptive tuberculosis: an individual participant data meta-analysis” (Submission PMEDICINE-D-25-00818R1). Hereby, we would like to submit our revised manuscript.

Please find below a point-by-point response to the comments raised. Where the text in the manuscript was amended, we highlighted the respective sections by underscoring.

**Comments from the academic editor:**

1) "Exclusion of participants with extra-pulmonary TB from analysis". Given the challenges of diagnosing all forms of TB in young children, and the high rate of extra-pulmonary TB, I struggle to see how the authors could have reliably done this. Additionally, I note that participants with LF-LAM positive urine were included in analysis, and this would usually indicate extra-pulmonary TB - needs clarification.

🡪 We appreciate this comment and agree to be clearer on the population. We excluded children solely diagnosed with extrapulmonary TB, such as TB meningitis or osteomyelitis, as reported by the local investigator. Children diagnosed with both intrathoracic as well as extrapulmonary manifestations were included. The section now reads the following:

*“We only included children with presumptive pulmonary tuberculosis (i.e., excluding those diagnosed by local investigators with sole extrapulmonary tuberculosis)…”*

2) Likewise, although mWRD positivity indicated treatment, it is not clear whether these tests were done on sputum, gastric aspirate, stool, or urine. Needs clarification, and specimen type may change case definition.

🡪 Sample collection varied across studies, but for the most part respiratory samples including spontaneous and induced sputum, nasopharyngeal aspirate, and gastric aspirate were analysed. Urine was not tested using mWRDs. Stool or urine results were not considered. We added the following to the methodology (outcome):

*“Microbiological testing was performed using XpertMTB/RIF or Ultra on respiratory samples, including spontaneous and induced sputum, nasopharyngeal aspirate, and gastric aspirate; stool results were not considered. Urine LAM was not considered as it was not standard-of-care during study conduct. The clinical case definitions… ”*

3) I didn't fully understand the analysis where 80% of "low risk" participants were removed from analysis to investigate triage testing approaches - I suspect that, given the considerable diagnostic uncertainty, this would not be a robust approach.

🡪 We initially included this additional sensitivity analysis as a thought experiment and to give an outlook on performance of the TDAs in a population with a large proportion of children that would not go to the scoring section because they would get better within 1-2 weeks. Hereby we wanted to stress that the possible impact on performance might be quite pronounced. Due to the scarcity of data on true TB prevalence as well as other diagnoses of children presenting to primary and secondary levels of care, we could not base the here-presented thought experiment on data and thus the focus is not to propose a statistical or methodological approach.

We appreciate that this should be better put in context and have therefore clarified the aim and implication accordingly. However, we are also happy to omit this section following the editor’s preference:

*“An additional sensitivity analysis aimed to model the triage step in the beginning of the TDA. Here, we wanted to explore the impact of having a large population of children with unlikely TB that would not present to health care again after initial evaluation. Due to scarcity of data on TB prevalence and other diagnoses of children presenting to primary and secondary care we randomly removed 80% of those who were considered low risk and ended up with an “unlikely tuberculosis” diagnosis.*”

4) As well as sensitivity and specificity estimates, important to show pooled estimates of positive and negative predictive values.

🡪 We have now included the pooled PPV and NPV for TDA A and B in the results

*“Pooled PPV and NPV were 63.0% and 75.4% for TDA A and 57.2% and 76.2% for TDA B.”*

5) Discussion is currently unreferenced, and needs greater critique of the limitations of the study.

🡪 We apologise and have now added the missing references in the discussion. We also have expanded the section on limitations:

*“As for limitations, although the study included high-level tuberculosis diagnostic studies and a large study conducted at primary health care levels, the heterogeneity of the studies led to uncertainty in the findings, though this improved generalisability. Most diagnostic tuberculosis studies in children, including two of those included in the IPD, are performed at higher levels of health care and thus the target population might differ somewhat from the IPD. In addition, because of the retrospective nature of the IPD evaluation, the “triage” steps prior to the microbiological, tuberculosis history, and scoring sections of the algorithm cannot be assessed, and these could modify overall sensitivity and specificity of the TDAs. Prospective studies are needed to see how the triage step impacts the diagnostic accuracy of these TDAs and to evaluate the feasibility and acceptability of the TDAs in practice. This also includes the potential development of similar tools for the use in active case finding among children without symptoms in future.”*

6) The authors suggest that using AI analysis of chest X-ray could improve specificity - I think the opposite is likely to be true - chest X-ray signs are generally non-specific for TB, and I suspect that AI algorithms will struggle to distinguish TB from non-TB findings, notwithstanding the fact that diagnostic accuracy CXR studies are very difficult to do in children. Addition of another low specificity test to a low-specificity algorithm will likely further worsen over-treatment. This proposal needs much greater consideration and supporting evidence.

🡪 AI analysis of chest X-ray in a disease with an imperfect reference standard are indeed cumbersome. Following the roll out of algorithms such as CAD4TB, efforts have been made to adapt/retrain those for paediatric purposes, but mostly to make this more accessible for settings where no expert reader is available. Palmer et al. demonstrated that following retraining, the area under the receiver operating characteristic curve (AUC) of the original CAD4TB model, prior to fine-tuning, was 0.58. After fine-tuning there was an improvement in the AUC to 0.72 (p = 0.0016; DOI: 10.1371/journal.pgph.0001799).

However, we agree with the reviewer that currently AI-tools do not outperform expert readers and will likely never do. Since this TDA is aimed to be used at the lowest levels of care by health care workers with very limited training to read and interpret CXRs. We discuss this in the following way:

*“However, it is important to consider that the CXR classifications included in this IPD were based on the interpretation of the CXR by tuberculosis experts, which might not reflect the CXR interpretation of less experienced readers. Furthermore, CXR can be challenging to interpret, costly to healthcare systems, and not all health workers are trained or accredited to read CXR for tuberculosis diagnosis. Computer-aided detection of CXR for the diagnosis of tuberculosis could overcome this barrier.”*

**Comments from the reviewers:**

**Reviewer #1: General comments**

This study retrospectively assessed the diagnostic accuracy of the 2022 WHO-recommended treatment decision algorithms for TB in children under 10 years of age using individual data from four studies. This is a well-conducted study that includes a large number of children and uses data from previous, rigorously conducted studies. The results reported are novel and important. The manuscript is clear and well-written.

1) My main comment is related to the fact that the results of the study inform about part of the algorithms (after what is called by the authors the "triage step") and not about the entire WHO-recommended algorithms. This is particularly relevant for the interpretation of the accuracy. Although this is mentioned in some parts of the manuscript, it is not stated in the abstract, and could be further highlighted in the introduction and the methods.

🡪 We agree and have changed the abstract to the following:

*“All children in the IPD were evaluated retrospectively using both TDA A (an algorithm including chest X-ray) and TDA B (without chest X-ray), excluding the triage step.*”

The introduction now reads:

*“In this study, we generate an individual participant dataset (IPD) derived from existing well-characterised paediatric TB cohorts to perform a retrospective external evaluation of the WHO TDAs, excluding the triage step.”*

The methods have been clarified:

*“Considering that this is a retrospective evaluation for the assessment of TDA performance in this study, we assumed all children progressed through the algorithm to the scoring part, i.e., excluding the triage step.”*

**Abstract**

2) I suggest stating in the introduction section and/or in the conclusions that the evaluation of the algorithms and the findings concern only part of the WHO-recommended algorithms (and not the entire WHO-recommended algorithms).

🡪 We agree and have changed accordingly, see response to comment above.

3) I suggest adding in the methods paragraph that children were "retrospectively" evaluated using both TDA A and TDA B.

🡪 It now reads:

*“All children in the IPD were retrospectively evaluated using both TDA A (an algorithm including chest X-ray) and TDA B (without chest X-ray), excluding the triage step. ”*

4) I think the last sentence in the conclusion is not clear. It is stated that "prospective studies should aim to enhance the specificity of these TDAs". Should this read that "prospective studies should assess the specificity of these TDAs"?

🡪 We rephrased to read the following:

*“… Prospective studies that evaluate the entire TDA, including triage step are needed. Additionally, the integration of novel diagnostic tools within the TDAs should aim to enhance the accuracy, especially the specificity.”*

**Methods**

5) Cohort level description: I suggest including in the description that the Umoya cohort excluded children who had an alternative diagnosis at baseline. This information is important as treating non-TB conditions is one of the steps in the WHO-recommended algorithms.

I also suggest including in the supplemental table 1 the definition of presumptive TB used in each of the four studies, and if possible, a summary of these definitions in the body of the manuscript.

🡪 The Umoya cohort included all children presenting with presumptive TB. In the end some ended up having an alternative diagnosis but they are part of this cohort. At screening level, children with an already established alternative diagnosis or pre-existing condition were not considered eligible, however, if pulmonary TB was still considered they were included.

For the definition of presumptive TB, we have adapted the Supplemental Table 1. For ease of reading we have briefly summarized this, but refer to the respective protocol papers for more details. In the manuscript, we now state the following:

*“Presumptive TB was defined similarly across the studies (Supplemental Table 1).* ”

6) Treatment Decision Algorithms: This section mentions that the algorithms include a "triage step". I suggest adding what this step consists of and stating that this step was excluded from the evaluation, and that therefore, the findings do not concern the entire WHO-recommended algorithms.

🡪 We agree and have adjusted the methods to the following:

*“The triage step identifies children with low risk of disease progression (i.e., >2 years of age, not living with HIV, and not being SAM), which are recommended to be treated for most likely non-TB conditions and followed up in 1-2 weeks. Considering that this is a retrospective...”*

7) Outcome: Can the authors clarify whether each participant was classified in a diagnostic category for the meta-analysis or if the diagnostic categorization by the primary studies was used?

🡪 We clarified in the methods:

“*The diagnostic categorisation or primary outcome was defined following the updated clinical case definition for classification of intrathoracic tuberculosis in children (NIH case definition). While we primarily used the definition of the diagnostic categorisation of the primary studies, these were aligned as much as possible. ”*

**Discussion**

8) Paragraph strengths and limitations: minor comment, consider stating that the limitations start at the beginning of the sentence where the first limitation is mentioned.

🡪 We agree and have adapted accordingly:

“As for limitations, although the study included high-level tuberculosis diagnostic studies…”

**Reviewer #2: PMEDICINE-D-25-00818R1**

General comments: The authors performed a retrospective external evaluation of WHO Treatment Decision Algorithms (TDAs) for childhood TB, using an individual participant dataset. The WHO TDAs were recommended by WHO in 2022, conditional on validation in different cohorts and settings. Development of the WHO TDAs was originally based on meta-analysis of a large individual participant dataset, using a previously published composite reference standard for Confirmed and Unconfirmed TB (Graham et al, CID 2015).

The main findings can be summarized by the Take Home Message: "When the WHO treatment-decision algorithms for children with presumptive tuberculosis were applied to an independent retrospective individual participant dataset, the performance was similar to that on the discovery dataset, with similar performance in vulnerable populations. Specificity remained sub-optimal".

Although the methodology was sound and the goal of the study was achieved, it is not entirely clear how the findings might be useful. Since the majority of childhood TB is not confirmed microbiologically, the study "validates" one algorithm for unconfirmed TB against another algorithm for unconfirmed TB. The findings bring us no closer to improved diagnostics for paucibacillary childhood TB, given the lack of a reliable gold standard.

🡪 We agree with the reviewer that there is no reliable gold standard for childhood TB. However, the WHO has conditionally recommended TDAs for use in primary care and the aim of this study was to externally validate these TDAs using well-defined cohorts. These well-defined cohorts are as close as we can currently get to a reliable reference standard for childhood TB. With the external validation of the TDAs we have identified potential weaknesses for implementation into primary care, which can lead to improvement in future.

**Abstract**

1) The abstract conclusion refers to high sensitivity, but suboptimal specificity of both TDAs. This is hardly surprising since the TDAs were designed around these parameters (see below). Suggest conclude that the observed performance was similar to that of the discovery dataset from which the TDAs were derived.

🡪 We agree with the reviewer and have added the following:

*“This retrospective external evaluation of WHO TDAs in a large IPD shows high sensitivity but sub-optimal specificity for both TDAs, in line with the meta-analyses that generated the algorithms. ”*

**Introduction.**

2) The need for sensitive diagnostics for childhood TB is discussed, but the need for more specific diagnostics should also be noted, given the findings and limitations below.

🡪 This is an important note. For now, overtreatment is widely accepted in the light of the high TB-associated mortality due to missed diagnosis, especially in young children. This is not without caveat and there is increasing awareness to identify measures to increase specificity. Within a tool such as the TDA, one possibility would be to add novel tools either for all children or specific subgroups. One of these tools with high potential is the 3-gene host response signature, which has been translated to a testing platform using fingerstick blood. In a previous study conducted in the RaPaed-TB cohort it has shown to have a high specificity of 90.3% (95% CI 85.5–94.0) while being moderately sensitive (59.8%, 95% CI 50.8–68.4), compared to culture, highlighting its potential to increase the specificity of a TDA, if included.

To address this comment, we have added the following in the manuscript:

*“The low overall specificity is concerning, especially in light of the imperfect reference standard, potentially leading to an overestimation in our dataset. In future, the addition of novel tools with high specificity, such as a three-gene host-response signature previously described to have a high specificity of 90.3% (95% CI 85.5–94.0) while being moderately sensitive (59.8%, 95% CI 50.8–68.4), might increase overall accuracy and especially specificity.”*

3) Suggest note in the Introduction the sensitivity and specificity of the scoring systems in the original TDA prediction models, which included CXR (86% sensitivity and 37% specificity), or only clinical features (84% sensitivity and 30% specificity), per Gunasekera et al, Lancet Adolesc Health 2023.

🡪 We appreciate this feedback and included the following in the introduction:

*“Based on a meta-analysis including 4,718 children from 13 studies from 12 countries, two TDAs were derived using prediction modelling. One scoring system was derived using clinical and radiological features with a combined sensitivity of 0.86 [95% CI 0.68-0.94] and specificity of 0.37 [0.15-0.66] against a composite reference standard. A second scoring system used only clinical features and had a combined sensitivity of 0.84 [95% CI 0.66-0.93] and specificity of 0.30 [0.13-0.56] against a composite reference standard. Following the generation of these and other evidence-based TDAs, the World Health Organization (WHO)...”*

**Methods**

The dataset is large, representative and fit for purpose.

**Results**

Diagnostic accuracy of the TDAs against the composite reference standard is reported as pooled sensitivities of 84.3% and 90.6%, and pooled specificities of 50.6% and 30.8%, for TDA A (with CXR) and B (clinical features only), respectively. As noted above, this performance is similar to that of the discovery IPD analysis (Gunasekera et al).

4) It would be useful to describe the characteristics of the group of false-negative TB cases that were missed, and who would not be treated by application of the TDAs; similarly, to describe the group of false-positive TB cases that would be treated unnecessarily by application of the TDAs.

🡪 We agree that including a description of those groups suffering from under- and overtreatment by both TDAs will be of interest and have thus included the following:

*“Based on TDA A, 28.7% (542/1,886) of children would have been overtreated, i.e., falsely deemed eligible for TB treatment, especially those at low risk 34.4% (357/1,039) and those older than 2 years of age (35.9% [197/549] and 30.5% [182/596]). Undertreatment was less common with 7.3% (138/1,886) of children not being eligible for TB treatment, particularly those living with HIV (14.9% [57/382]). For TDA B, patterns for overtreatment were similar with 40.9% (425/1,039) children at low risk and children older than 2 years (43.5% [239/549] and 36.7% [219/596]) being incorrectly classified as eligible. Undertreatment occurred in 4.5% (885/1,886) of children overall, particularly in those living with HIV (7.3% [28/382]).”*

5) The main text Figure legends appear to missing from the file.

🡪 We apologise have now added these to the main manuscript file, following the references.

**Discussion**

6) The main limitation of the study is the same as that of the original TDA discovery study, ie. uncertainty of diagnosis of unconfirmed TB included in the composite reference standard. The authors refer to "sub-optimal specificity" against this composite reference standard, but true specificity is probably lower. It would be useful to note the implications of this limitation for TDA false-positive diagnosis, unnecessary treatment burden, and treatment-related adverse effects.

🡪 The reviewer highlights an important limitation, which hampers all paediatric TB research, being the uncertainty around diagnostic classification and reference standards. Thus, we have included the following in the limitations section:

*“Despite the considerable effort and resources that went into these mostly diagnostic studies, a major inherent limitation is the uncertainty of true TB status of children without microbiological confirmation, resulting in an unclear estimation of diagnostic performance of TDAs. ”*

We also have added the following to the discussion on implications of over treatment:

*“The low overall specificity is concerning, especially in light of the imperfect reference standard, potentially leading to an overestimation in our dataset.“*

7) Some discussion of the group characteristics of TDA false-negative and TDA false-positive TB cases would provide useful insight into the consequences of under- and over-treatment.

Following the adaptation of the results, we have added the following to the discussion:

*“This tendency of some overtreatment with the TDAs was evident when comparing the TDAs to the local clinician’s decision to treat. Among the children classified as unlikely TB, over 50% of children recommended for treatment by the TDAs were not treated by the local (study) clinician. Overtreatment was particularly common in children with low risk and those older than 2 years, where around a third were falsely identified as eligible for treatment. In contrast, undertreatment was low overall. It is important to note, however, that these study clinicians are generally very experienced in diagnosing tuberculosis in children…”*

8) A limitation of this analysis, and of the datasets used to generate the TDAs, and of the composite reference standard, is that they include only symptomatic children presenting with presumptive pulmonary TB. Suggest note as a limitation that the findings are not generalizable to active case-finding among children without symptoms who have not presented for care.

🡪 The reviewer makes an interesting point. Currently, the available TDAs were developed for the use in symptomatic children presenting at lower levels of health care. Similar approaches could be developed in future to support active case findings. We have included the following:

*“This also includes the potential development of similar tools for the use in active case finding among children without symptoms in future.”*

**Reviewer #3**

The authors conducted a pooled analysis of four well-recognized studies in childhood TB to retrospectively calculate the accuracy of the WHO treatment decision algorithms. This is an important effort given the conditional recommendation, and can guide current use and directions for improvement including the need for higher specificity.

**MAJOR**

1) Abstract - would clarify that the accuracy is being measured after the triage phase/at the point of testing and clinical evaluation

🡪 Thanks for the suggestion, we agree and have added this as also suggested by the other reviewer. We have changed the abstract to the following:

*“All children in the IPD were evaluated using both TDA A (an algorithm including chest X-ray) and TDA B (without chest X-ray), excluding the triage step.*”

2) Abstract - please add the accuracy when stratified by high vs. low risk children

🡪 We have amended the abstract following the suggestion:

*“For TDA A, estimated sensitivity in children in high-risk groups was lower than those with low-risk (83.0%, 95%CI: 79.4% - 86.1%; vs 88.0%, 95%CI: 84.8% - 90.6%), while having a gain in specificity (50.0%, 95%CI: 44.9% - 55.1%; vs 36.6%, 95%CI: 32.7% - 40.7%). Trends were similar for TDA B.”*

3) Overall it is not clear at which point the accuracy is being measured at the testing/evaluation phase. The total sample size in the IPD analysis is 1,886, but then there is a focus on the scoring part (page 6 "in this study, we assumed all children progressed through the algorithm to the scoring part"). Is this the accuracy if all children were scored, or accuracy if the TDA was positive (i.e. +molecular testing, contact, or score >10)? Please clarify in methods/results.

🡪 We have tried to clarify this in a previous comment by defining the triage step:

*“Of the 1,886 children, we assumed all children progress beyond the initial triage step, treating children with a low risk for disease progression for non-TB illnesses and follow-up 1-2 weeks later. In total, 1,831 (97.1%) had a molecular WHO-recommended rapid diagnostic (mWRD) test result (Xpert MTB/RIF) performed on respiratory samples. Of those, 202 (11.0%) were positive and thus immediately eligible for treatment initiation (Figure 1). Of the 1,684 who did not have a positive molecular microbiological test result (negative or not done), 814 (48.3%) reported having close or household TB exposure (no time restriction) so were recommended treatment initiation, leaving 870 children eligible for the scored section of either TDA A or B. In total 400 (46.0%) and 557 (64.0%) of the children scored >10 for TDA A and B, respectively, and thus treatment initiation would have been recommended. Study-specific cascades can be found under Supplemental Figures 1 (TDA A) and 2 (TDA B).*”

4) TB exposure was not considered to be in the scoring part, but actually it is part of the scoring; in the meta-analysis (PMID: 36924781), TB contact had a score >10 and therefore was moved out of the box, and is part of the model used to calculate the other scores. Thus, would include it when considering the accuracy and score distribution.

🡪 We appreciate the reviewer highlighting this important point. Indeed, the initial meta-analysis included TB contact in the scoring section, however, the WHO TDAs do not. While these were developed based on the work of Gunasekera et al., they were adapted during the review process. As our study here aims to evaluate the WHO TDAs we structured our analysis accordingly.

We have amended the introduction to read the following

*“Following the generation of these and other evidence-based TDAs, the World Health Organization (WHO) issued a conditional recommendation for the use of TDAs in children under ten years with presumptive pulmonary tuberculosis and included two exemplar TDAs adapted from the above-mentioned meta-analysis in their 2022 operational handbook.”*

5) At least for this reviewer, there does not appear to be figure titles or legends, and Figure 3 is mislabeled as Figure 2 in the scoring section of results.

🡪 We apologise for this and have both included the legends as well as corrected the labelling.

6) Figure 2 overall is not clear, do the each bar represent proportion positive? I also assume no TB and TB are based on the CRS, but this makes the clinician only interpretation challenging as their decisions influence the definition of Unconfirmed TB.

🡪 We agree with the reviewer there is some incorporation bias when comparing NIH TB case definitions and clinicians decision to treat. This is an inherent issue when using the NIH classification, we have updated the discussion of limitations to the following:

*“Most diagnostic tuberculosis studies in children, including two of those included in the IPD, are performed at higher levels of health care and thus the target population might differ somewhat from the IPD. Despite the considerable effort and resources that went into these studies, a major inherent limitation is the uncertainty of true TB status of children without microbiological confirmation, resulting in an unclear estimation of diagnostic performance of TDAs. This also includes potential incorporation bias, for example as there is an overlap between the reference standard definition (i.e., unconfirmed TB) and TDA scoring. In addition, because of the retrospective nature… ”*

We have changed the labelling of the figure.

7) "There was some concordance" (Page 12). Please quantify the overall concordance and discordance

🡪 We have changed as suggested:

*“There was reasonable concordance (85.4% TDA A, 79.9% TDA B) between the local clinician's decision-to-treat before implementation of the TDA and the retrospective TDA-based recommendation, though for both TDAs among children with unlikely TB, there was a substantial (52% TDA A, 63% TDA B) group of children who would have been started on treatment based on the TDA but for whom treatment was not initiated by the local and/or study clinicians (Figure 2). “*

8) Figure 3 - if excluded those with negative/no microbiological findings, then by definition this is looking at Unconfirmed vs. Unlikely TB, but the text (page 12) notes Confirmed or Unconfirmed? How can those with Confirmed TB then have a median score?

🡪 Thank you for this important comment. You are right this analysis does not include the children with confirmed TB. We have amended the text to reflect this and have added a clarification in the figure footnote.

*“Among those who were eligible for the scoring part of the TDA (i.e., negative/no microbiological findings and no history of TB contact), children with confirmed or unconfirmed TB tended to have higher median scores than children with unlikely TB (TDA A: confirmed 12, IQR:6-23, unconfirmed 12, IQR:7-18, unlikely 7, IQR:3-11; TDA B: confirmed 15, IQR:7-23, unconfirmed 16, IQR:9-24, unlikely 11, IQR:5-16) (Figure 3).”*

9) Figure 3 - I think we would expect higher scores given the overlap between the scoring system and having Unconfirmed TB? Given the scores are higher, is there a higher score threshold that could be then used to increase specificity without significant loss of sensitivity? The implication and limitations of these findings should be in the discussion.

🡪 We amended the implications to the following:

*Prospective evaluations to allow assessment of the entire algorithm, including the triage step are an important next step. This also includes to explore different thresholds for the scoring section, especially in subgroups of interest, which may also be dependent on settings, symptoms, and other characteristics. It will also be important to combine these TDAs with novel diagnostic tools, including biomarkers and AI-based tools, or sampling strategies to further improve TDA specificities, improve efficiency and ensure their feasibility for use in lower levels of care.*

10) There will be incorporation bias with the CRS as the definitions for Confirmed and Unconfirmed TB include microbiological testing, and there is overlap in the scoring and Unconfirmed definition. Please discuss this limitation and the implications in the discussion.

🡪 The reviewer highlights an important limitation of paediatric TB research. Having an imperfect reference standard, i.e., microbiological testing, leads to a number of methodological challenges. We have updated the discussion of limitations to the following:

*“Most diagnostic tuberculosis studies in children, including two of those included in the IPD, are performed at higher levels of health care and thus the target population might differ somewhat from the IPD. Despite the considerable effort and resources that went into these studies, a major inherent limitation is the uncertainty of true TB status of children without microbiological confirmation, resulting in an unclear estimation of diagnostic performance of TDAs. This also includes potential incorporation bias, for example as there is an overlap between the reference standard definition (i.e., unconfirmed TB) and TDA scoring. In addition, because of the retrospective nature… ”*

11) LAM testing is also part of the algorithm among children with HIV, but not mentioned, please include or note as a limitation

🡪 This was done in RaPaed-TB but not standard in the other studies as that was not part of standard of care yet, thus we have not included this. We have added this to the methods and limitations:

*“Microbiological testing was performed using XpertMTB/RIF or Ultra on respiratory samples, including spontaneous and induced sputum, nasopharyngeal aspirate, and gastric aspirate; stool results were not considered. Urine LAM was not considered as it was not standard-of-care during study conduct. The clinical case definitions… ”*

*“As for limitations, although the study included high-level tuberculosis diagnostic studies and a large study conducted at primary health care levels, the heterogeneity of the studies led to uncertainty in the findings, though this improved generalisability. During study conduct, urine LAM-testing was not standard-of-care and thus not considered here.* ”

**MINOR**

12) Table 1 - could you add the proportion that has normal CXRs?

🡪 We have amended the table to include the proportion of normal CXR by study.

13) For the analysis where 80% of low-risk children with Unlikely TB are excluded, clarify if this accuracy is overall or just for the low-risk children

🡪 We have added the following:

*“Removing 80% of the low risk children who were classified as unlikely TB improved both specificity (TDA A: 51.1% [95%CI:33.5-68.3], TDA B: 33.7% [95%CI:26.2-42.2]) and sensitivity (TDA A: 84.3% [95%CI:74.8-90.6], TDA B: 90.7% [95%CI:84.1-94.7]) in the overall cohort.”*

14) "In addition, the treatment of tuberculosis infection (using 2 drugs for 3 months) and non-severe disease (using 3-4 drugs for 4 months) are fairly similar which limits the additional burden to have a low threshold of initiating treatment in these children." This is an oversimplification of the differences in TB infection and disease management, and associated other challenges including missed alternative diagnoses, stigma, isolation, costs, etc. While the importance of empiric treatment is noted, would suggest still emphasizing the authors' main point that greater specificity is still needed.

🡪 We agree with the reviewers suggestion and have removed the suggested sentence:

*~~”In addition, the treatment of tuberculosis infection (using 2 drugs for 3 months) and non-severe disease (using 3-4 drugs for 4 months) are fairly similar which limits the additional burden to have a low threshold of initiating treatment in these children”~~*

We have replaced this by:

*“The low overall specificity is concerning, especially in light of the imperfect reference standard, potentially leading to an overestimation in our dataset. In future, the addition of novel tools with high specificity, such as a three-gene host-response signature previously described to have a high specificity of 90.3% (95% CI 85.5–94.0) while being moderately sensitive (59.8%, 95% CI 50.8–68.4), might increase overall accuracy and especially specificity.”*
