# Supplementary material for: Diagnostic accuracy of the WHO tuberculosis treatment decision algorithms for children with presumptive tuberculosis: An individual participant data meta-analysis
Source: PLoS Med. 2025 Nov 18;22(11):e1004610. doi: 10.1371/journal.pmed.1004610 (PMC12626314; doi:10.1371/journal.pmed.1004610)
Supplement: Attachment — Submitted filename: ResponseToComments_20250916.docx [file pmed.1004610.s005.docx]

**Response to comments, 2^nd^ revision**

Dear Editor and reviewers,

We are delighted with the overall acceptance of the paper and the chance to make the final revisions. Please find below a step by step response.

Best wishes,

Marieke van der Zalm on behalf of all authors.

We ask every co-author listed on the manuscript to fill in a contributing author statement. If any of the co-authors have not filled in the statement, we will remind them to do so when the paper is revised. If all statements are not completed in a timely fashion this could hold up the re-review process. Should there be a problem getting one of your co-authors to fill in a statement we will be in contact. YOU MUST NOT ADD OR REMOVE AUTHORS UNLESS YOU HAVE ALERTED THE EDITOR HANDLING THE MANUSCRIPT TO THE CHANGE AND THEY SPECIFICALLY HAVE AGREED TO IT.

**🡪 noted**

Please ensure that the paper adheres to the PLOS Data Availability Policy (see [https://zaf01.safelinks.protection.outlook.com/?url=http%3A%2F%2Fjournals.plos.org%2Fplosmedicine%2Fs%2Fdata-availability&data=05%7C02%7C%7Ccb2a20c5f25242df58b508ddefe2cfa9%7Ca6fa3b030a3c42588433a120dffcd348%7C0%7C0%7C638930479649004286%7CUnknown%7CTWFpbGZsb3d8eyJFbXB0eU1hcGkiOnRydWUsIlYiOiIwLjAuMDAwMCIsIlAiOiJXaW4zMiIsIkFOIjoiTWFpbCIsIldUIjoyfQ%3D%3D%7C0%7C%7C%7C&sdata=SmjrcQWIaGJIUbodKV%2Fbxe6azILbiO19SbpdF%2FXz6rM%3D&reserved=0)](http://journals.plos.org/plosmedicine/s/data-availability), which requires that all data underlying the study's findings be provided in a repository or as Supporting Information. For data residing with a third party, authors are required to provide instructions with contact information for obtaining the data. PLOS journals do not allow statements supported by "data not shown" or "unpublished results." For such statements, authors must provide supporting data or cite public sources that include it.

- **“Data Availability Statement**
  The IPD will be made available upon reasonable request with investigator support and a signed data access agreement.”

GENERAL EDITORIAL REQUESTS

* Please use the active voice throughout.

* At this stage, we ask that you include a short, non-technical Author Summary of your research to make findings accessible to a wide audience that includes both scientists and non-scientists. The Author Summary should immediately follow the Abstract in your revised manuscript. This text is subject to editorial change and should be distinct from the scientific abstract. Ideally each sub-heading should contain 2-3 single sentence, concise bullet points containing the most salient points from your study. In the final bullet point of ‘What Do These Findings Mean?’ please include the main limitations of the study in non-technical language.

- **Author summary**

***Why was this study done?***

- Tuberculosis in children remains one of the top ten causes of death in those younger than five years, mainly due to missed or delayed diagnosis. This is especially challenging in primary health care settings, where available tests are difficult to perform, require substantial infrastructure, and lack sufficient accuracy.
- In 2022, the WHO recommended treatment decision algorithms for TB, which are simple flow-charts designed to guide healthcare workers step by step through a standardized diagnostic process that relies primarily on clinical information. These algorithms aim to support and standardize treatment decision, but evidence on their performance remains limited.

***What did the research find?***

- In our study, we used data from several large previously conducted studies on children that underwent testing for tuberculosis, to evaluate how good these treatment decision algorithms perform to identify children with tuberculosis.
- We found that the performance in this independent dataset of children was comparable to that reported in the original discovery study. While the algorithms identified a large number of children with tuberculosis (high sensitivity), it also recommended to start a considerable number of children without tuberculosis on treatment (sub-optimal specificity).
- The accuracy was similar also in those children of vulnerable populations, including young children and those affected by HIV or malnutrition.

***What do the findings mean?***

- This is the first study to use previously collected data from several studies to assess how accurate the WHO treatment decision algorithms are. It shows that the estimated performance is similar to the actual one, importantly also in vulnerable populations.
- Our findings underscore the potential usefulness of diagnostic approaches such as treatment decision algorithms to identify more children eligible for tuberculosis treatment. By using a tool that can potentially be implemented at low levels of health care, this approach might help to avert many deaths due to childhood tuberculosis.

* Please confirm that your abstract complies with our requirements, including format (three sections: Background, Methods and Findings, and Conclusions) and providing all the information relevant to this study type [https://zaf01.safelinks.protection.outlook.com/?url=https%3A%2F%2Fjournals.plos.org%2Fplosmedicine%2Fs%2Fsubmission-guidelines%23loc-abstract&data=05%7C02%7C%7Ccb2a20c5f25242df58b508ddefe2cfa9%7Ca6fa3b030a3c42588433a120dffcd348%7C0%7C0%7C638930479649047697%7CUnknown%7CTWFpbGZsb3d8eyJFbXB0eU1hcGkiOnRydWUsIlYiOiIwLjAuMDAwMCIsIlAiOiJXaW4zMiIsIkFOIjoiTWFpbCIsIldUIjoyfQ%3D%3D%7C0%7C%7C%7C&sdata=T5Wzg9ZhyPq4dzXh4rEuQfyRqQ6Rnb%2Fg7KqS7Iip7B8%3D&reserved=0](https://journals.plos.org/plosmedicine/s/submission-guidelines#loc-abstract)

- **Done**

* Please ensure that the Introduction ends with a clear description of the study question or hypothesis.

- **Done**

* Please use continuous line numbering. Do not restart numbering on each page.

- **Done**

* Please ensure that where relevant figures include 95% CIs. Please use commas in confidence intervals, throughout.

- **This is not required in any of the figures.**

* Please ensure that all abbreviations are defined at first use throughout the text.
* Please confirm that all numbers presented in the abstract are present and identical to numbers presented in the main manuscript text.
* In the last sentence of the Abstract Methods and Findings section, please describe the main limitation(s) of the study's methodology.

- **Done**

* In the abstract, please include the important dependent variables that are adjusted for in the analyses.

- **No analyses were conducted which had dependent variables as the analysis was primarily descriptive followed by a diagnostic accuracy analysis.**

*The Data Availability Statement (DAS) requires revision. For each data source used in your study:
a) If the data are freely or publicly available, note this and state the location of the data: within the paper, in Supporting Information files, or in a public repository (include the DOI or accession number).

b) If the data are owned by a third party but freely available upon request, please note this and state the owner of the data set and contact information for data requests (web or email address). Note that a study author cannot be the contact person for the data.

- **Done, see above**

c) If the data are not freely available, please describe briefly the ethical, legal, or contractual restriction that prevents you from sharing it. Please also include an appropriate contact (web or email address) for inquiries (again, this cannot be a study author).

* If new code was created for this analysis, please include the statement on code availability in the data availability statement.

- **The GitHub link was added to the data-availability section.**

* Please specify whether consent in the relevant studies was informed consent  and whether it was written or oral. Please ensure that the research complies with the PLOS policy in full: [https://zaf01.safelinks.protection.outlook.com/?url=https%3A%2F%2Fjournals.plos.org%2Fplosmedicine%2Fs%2Fhuman-subjects-research%23loc-patient-privacy-and-informed-consent-for-publication&data=05%7C02%7C%7Ccb2a20c5f25242df58b508ddefe2cfa9%7Ca6fa3b030a3c42588433a120dffcd348%7C0%7C0%7C638930479649061574%7CUnknown%7CTWFpbGZsb3d8eyJFbXB0eU1hcGkiOnRydWUsIlYiOiIwLjAuMDAwMCIsIlAiOiJXaW4zMiIsIkFOIjoiTWFpbCIsIldUIjoyfQ%3D%3D%7C0%7C%7C%7C&sdata=VwcVNBvzs0pB8WZ%2F0i2K3r2rqVvYO2qMNvpdNfTUaYo%3D&reserved=0](https://journals.plos.org/plosmedicine/s/human-subjects-research#loc-patient-privacy-and-informed-consent-for-publication)

- **Done**

* Please clarify whether this study received approval from a specific IRB. If yes, please identify if in the Methods.

- **Done, see above**

* Figures cannot be reproduced from other sources that are not CC-BY. Please clarify whether this applies to any of the figures, including in SI. Please clarify whether any figures are from the WHO.

- **All figures which were reproduced were from the WHO operational handbook which has a CC-BY-NC license and hence under the terms of this licence, one may copy, redistribute and adapt the work for non-commercial purposes, provided the work is appropriately cited, as we have done.**

* Please define all acronyms used in each figure or table in its corresponding legend.

- **Done**

*Please specify the variables controlled for in all relevant Tables

- **NA**

* In the last sentence of the Abstract Methods and Findings section, please describe the main limitation(s) of the study's methodology.

- **Done**

* In the abstract, please include the important dependent variables that are adjusted for in the analyses.

- **No analyses were conducted which had dependent variables as the analysis was primarily descriptive followed by a diagnostic accuracy analysis.**

First sentence in section TDA cascade: phrasing is awkward, please revise.

🡪 **we have rephrased this as suggested.**

FUNDING STATEMENT
* The funding statement should include: specific grant numbers, initials of authors who received each award, and URLs (currently missing) to sponsors’ websites. Also, please state whether any sponsors or funders (other than the named authors) played any role in study design, data collection and analysis, the decision to publish, or preparation of the manuscript. If they had no role in the research, include this sentence: “The funders had no role in study design, data collection and analysis, decision to publish, or preparation of the manuscript.”

- **Done**

For Observational studies:

* Please ensure that the study is reported according to the STROBE guideline, and include the completed STROBE checklist as Supporting Information.  Please add the following statement, or similar, to the Methods: "This study is reported as per the Strengthening the Reporting of Observational Studies in Epidemiology (STROBE) guideline (S1 Checklist)."
The STROBE guideline can be found here: [https://zaf01.safelinks.protection.outlook.com/?url=http%3A%2F%2Fwww.equator-network.org%2Freporting-guidelines%2Fstrobe%2F&data=05%7C02%7C%7Ccb2a20c5f25242df58b508ddefe2cfa9%7Ca6fa3b030a3c42588433a120dffcd348%7C0%7C0%7C638930479649075286%7CUnknown%7CTWFpbGZsb3d8eyJFbXB0eU1hcGkiOnRydWUsIlYiOiIwLjAuMDAwMCIsIlAiOiJXaW4zMiIsIkFOIjoiTWFpbCIsIldUIjoyfQ%3D%3D%7C0%7C%7C%7C&sdata=Kaunp9lVwRy5xn0j63NlQy49UY5CIE4s8IX84CKOqno%3D&reserved=0](http://www.equator-network.org/reporting-guidelines/strobe/)
When completing the checklist, please use section and paragraph numbers, rather than page numbers.

- **Not applicable**

* Did your study have a prospective protocol or analysis plan? Please state this (either way) early in the Methods section.

a) If a prospective analysis plan (from your funding proposal, IRB or other ethics committee submission, study protocol, or other planning document written before analyzing the data) was used in designing the study, please include the relevant prospectively written document with your revised manuscript as a Supporting Information file to be published alongside your study, and cite it in the Methods section. A legend for this file should be included at the end of your manuscript.
b) If no such document exists, please make sure that the Methods section transparently describes when analyses were planned, and when/why any data-driven changes to analyses took place.
c) In either case, changes in the analysis-- including those made in response to peer review comments-- should be identified as such in the Methods section of the paper, with rationale.

- **Done**

* For all observational studies, in the manuscript text, please indicate: (1) the specific hypotheses you intended to test, (2) the analytical methods by which you planned to test them, (3) the analyses you actually performed, and (4) when reported analyses differ from those that were planned, transparent explanations for differences that affect the reliability of the study's results. If a reported analysis was performed based on an interesting but unanticipated pattern in the data, please be clear that the analysis was data-driven.

- **Done**

**Comments from Reviewers:**

Reviewer #1: Thanks to the authors for their answers and the modifications in the manuscript. All my comments have been addressed. I do not have any additional comments.

- **We appreciate the feedback.**

Reviewer #2: The authors have added explanatory text and substantially improved the manuscript in terms of clarity, accuracy and interpretation.

I have one minor point re. the new sentence (page 14, lines 22-25) "In future, the addition of novel tools with high specificity, such as a three-gene host-response signature previously described to have a high specificity of 90.3% (95% CI 85.5- 94.0) while being moderately sensitive (59.8%, 95% CI 50.8-68.4), might increase overall accuracy and especially specificity."

While I agree with the sentiment that in future, the addition of novel diagnostic tools might increase overall accuracy and especially specificity of the TDAs, I am not sure that reference #19 (Olbrich et al) is accurate for this purpose, since the quoted specificity of 90.3% and sensitivity of 59.8% is for discrimination of Confirmed vs Unlikely TB.

If the authors wish to quote this reference to support use of the 3-gene signature in the TDAs they should quote the specificity of 90.3% and sensitivity of 29.6% for discrimination of Confirmed and Unconfirmed vs Unlikely TB. Alternatively, since sensitivity is sub-optimal, a more generic statement would be appropriate.

- **As suggested, we have removed this reference and text.**

Reviewer #3: The paper is clearer and they authors have appropriately addressed the comments. One minor comment is that in Figure 3 on the distribution of scores, for High/Low Risk, the plots are stratified into "Yes" and "No". I would suggest you instead label it as "High Risk" or "Low Risk," which would clarify the plots and would be align with the rest of the figure.

- **Done**
